# Persistent Hypertension Up to One Year Postpartum among Women with Hypertensive Disorders in Pregnancy in a Low-Resource Setting: A Prospective Cohort Study

**DOI:** 10.5334/gh.854

**Published:** 2021-09-09

**Authors:** Salisu M. Ishaku, Tukur Jamilu, Agbo P. Innocent, Kayode A. Gbenga, Dattijo Lamaran, Oyeneyin Lawal, Charlotte E. Warren, Owa O. Olorunfemi, Hanifah D. Abubakar, Tunau Karima, Odusolu O. Patience, Abdulkarim Musa, Onyebuchi K. Azubuike, Aminu M. Baffah, Arie Franx, Diederick E. Grobbee, Joyce L. Browne

**Affiliations:** 1 Population Council Nigeria, NG; 2 Julius Global Health, Julius Center for Health Science and Primary Care, UMC Utrecht, Utrecht University, NL; 3 Bayero University/Aminu Kano Teaching Hospital Kano, Kano State, NG; 4 Institute of Human Virology, Abuja, NG; 5 Abubakar Tafawa Balewa University Teaching Hospital, Bauchi, Bauchi State, NG; 6 University of Medical Sciences Teaching Hospital, Ondo, Ondo State, NG; 7 Population Council-Washington, DC, US; 8 Mother and Child Hospital, Akure, NG; 9 Muhammad Abdullahi Wase Teaching Hospital, Kano, Kano State, NG; 10 Usman DanFodio University Teaching hospital, Sokoto, Sokoto State, NG; 11 University of Calabar Teaching Hospital, Calabar, Cross River State, NG; 12 Federal Medical Center, Lokoja, Kogi State, NG; 13 Federal Teaching Hospital, Abakaliki, Ebonyi State, NG; 14 Erasmus Medical Center, University Medical Center Rotterdam, NL

**Keywords:** Hypertensive Disorder in Pregnancy, Persistent Hypertension, Nigeria

## Abstract

**Background:**

Hypertensive disorders in pregnancy (HDPs) are associated with lifelong cardiovascular disease risk. Persistent postpartum hypertension in HDPs could suggest progression to chronic hypertension. This phenomenon has not been well examined in low- and middle-income countries (LIMCs), and most previous follow-ups typically last for maximally six weeks postpartum. We assessed the prevalence of persistent hypertension up to one year in women with HDPs in a low resource setting and determined associated risk factors.

**Methodology:**

A prospective cohort study of women conducted at eight tertiary health care facilities in seven states of Nigeria. Four hundred and ten women with any HDP were enrolled within 24 hours of delivery and followed up at intervals until one year postpartum. Descriptive statistics were performed to express the participants’ characteristics. Univariable and multivariable logistic regressions were conducted to identify associated risk factors.

**Results:**

Of the 410 women enrolled, 278 were followed up to one year after delivery (follow-up rate 68%). Among women diagnosed with gestational hypertension and pre-eclampsia/eclampsia, 22.3% (95% CI; 8.3–36.3) and 62.1% (95% CI; 52.5–71.9), respectively, had persistent hypertension at six months and this remained similar at one year 22.3% (95% CI; 5.6–54.4) and 61.2% (95% CI; 40.6–77.8). Maternal age and body mass index were significant risk factors for persistent hypertension at one year [aORs = 1.07/year (95% CI; 1.02–1.13) and 1.06/kg/m^2^ (95% CI; 1.01–1.10)], respectively.

**Conclusion:**

This study showed a substantial prevalence of persistent hypertension beyond puerperium. Health systems in LMICs need to be organized to anticipate and maintain postpartum monitoring until blood pressure is normalized, or women referred or discharged to family physicians as appropriate. In particular, attention should be given to women who are obese, and or of higher maternal age.

## Introduction

Globally, Hypertensive disorders in pregnancy (HDPs) complicate 5–10% of pregnancies and annually responsible for, at least, 70,000 and 500,000 maternal and neonatal deaths, respectively [1,2]. The spectrum includes chronic hypertension (secondary or essential), gestational hypertension and (pre-)eclampsia [2]. In Nigeria, pre-eclampsia contributes to over 23% of the direct causes of maternal mortality in referral health facilities [3]. Bearing in mind delivery of the placenta is considered the cure for HDPs (except chronic hypertension), the traditional expectation is that hypertension resolves by the end of the puerperium, a period generally defined as six to eight weeks after delivery [4]. However, emerging evidence suggests that hypertension may persist for a variable period beyond puerperium and up to years after delivery – especially following severe forms of HDPs such as pre-eclampsia and eclampsia [5,6]. This is due to a combination of two factors. First, women with HDPs have an increased lifetime risk of future chronic hypertension, cardiovascular disease risk factors and cardiovascular diseases due to underlying vascular (re)consistitution or HDP-related (vascular) damage [7,8]. Second, women with previous undiagnosed chronic hypertension are misclassified during pregnancy as gestational hypertension [7,34,35], and their persisting hypertension beyond puerperium erroneously considered new onset chronic hypertension following HDPs.

Therefore, estimation and quantification of the true magnitude of persistent hypertension in HDPs is an important initial step to curb the risk of future chronic hypertension and adverse cardiovascular events [7,8], for which sub-Saharan Africa suffers disproportionately [9]. Despite these facts, few studies have been conducted in low-resource settings and mostly restricted to short postpartum follow up [10,11,12,13,14]. This study prospectively determined the prevalence of hypertension over one year in a cohort of Nigeria’s women with HDPs who delivered in tertiary facilities and assessed the risk factors associated with persistent hypertension.

## Methods

### Study design

The study was a prospective cohort study. Women with HDP were recruited from August 2017 to April 2018 and followed up for one year. The last woman recruited exited the study on March 31, 2019.

### Study setting

**Figure 1:**
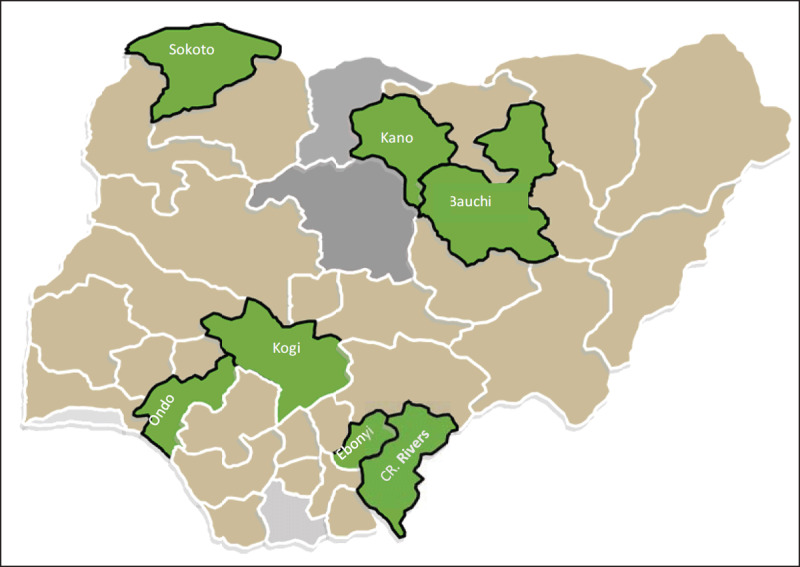
Map of Nigeria with the states in which the study sites were located.

The study was conducted at eight tertiary hospitals in the six geo-political zones of Nigeria (Figure 1). The hospitals were purposefully selected to reflect diversity in terms of ethnicity and socio-economic status and included: Bauchi State (Abubakar Tafawa Balewa University Teaching Hospital, ATBUTH), Cross River State (University of Calabar Teaching Hospital, UCTH), Ebonyi State (Federal Teaching Hospital Abakaliki, FTHA), Kogi State (Federal Medical Center, FMC Lokoja), Kano State (Aminu Kano Teaching Hospital, AKTH), Ondo State (Mother and Child Hospital Akure and University of Medical Sciences Teaching Hospital, Ondo) and Sokoto State (Usman Danfodio University Teaching Hospital, UDUS). Women who received care in these facilities were considered a good representation of the diversity in the country. The health facilities are high-volume and well-functioning sites with combined annual deliveries averaging 38,400.

### Participants

Women who delivered at the facilities (regardless of where they received their antenatal care), who were 18 years or above, and had a diagnosis of HDPs were eligible for inclusion. Exclusion criteria included: multiple pregnancies, medical disorders in pregnancy other than HDPs (e.g., diabetes mellitus, sickle cell disease, heart disease, kidney and other connective tissues disorders).

### Study procedures

All women with HDPs were informed of the study either during antenatal care period (for registered clients) or after delivery but all recruitments were done in the postpartum period. Those willing to participate were individually counseled, informed consent obtained – either signed or thumb-printed- (consent rate over 95%) and enrollment forms (a questionnaire that collected information on socio-demographic and obstetrics variables) completed within 24 hours of delivery. After enrollment, the women underwent baseline clinical evaluations to assess their symptoms and signs. The clinical evaluation involved general and systemic examination including the determination of the blood pressure measurements. In addition, laboratory investigations were performed on the participants before they were discharged from the hospitals. They were subsequently followed-up during which the same clinical and laboratory investigations conducted at baseline were repeated at nine weeks, six months and one year postpartum. To improve follow up rate over the entire duration of one year, the research participants were requested (if willing and consented) to provide their contact information (particularly personal and spousal mobile telephone numbers). They were reminded of their follow up appointments through phone calls. Participants’ contact information was not linked to their clinical records while all clinical information was linked to unique identifiers.

### Exposures variables

The main exposure of interest was the presence of any of the HDP sub-types including chronic hypertension, gestational hypertension and pre-eclampsia as defined by the International Society for the Study of Hypertension in Pregnancy (ISSHP) [1,2].

Hypertension was defined as systolic blood pressure of ≥140 mmHg and/or diastolic blood pressure of ≥90 mmHg measured on two consecutive periods 4–6 hours apart. Chronic hypertension in pregnancy was defined as any hypertension with onset before the index pregnancy or diagnosed within the first 20 weeks of the index pregnancy. Gestational hypertension was defined as any hypertension occurring after the first 20 weeks of pregnancy without significant proteinuria (<2++ of proteinuria on urine dipstick measurement) or any haematological or biochemical abnormality. Pre-eclampsia was defined as hypertension with onset after the first 20 weeks of pregnancy with significant proteinuria (≥2++ of proteinuria on urine dipstick measurement) or the presence of any haematological and biochemical abnormality.

### Outcome variables

The main outcome variable was persistent hypertension, defined as the occurrence of systolic blood pressure of ≥140 mmHg and/or, diastolic blood pressure of ≥90mmHg measured at least four to six hours apart after eight weeks postpartum. Other outcome measures of interest included persistent significant proteinuria after delivery (≥2++ of proteinuria on urine dipstick measurement), BMIs (weight[kg]/height[ m^2^]), high serum creatinine greater than 90 mmol/L, high serum urea greater than 7.1 mmol/L, high uric acid greater than 446 mmol/L, raised Alanine transaminase (ALT) above 55 units/liter, raised Aspartate transaminase (AST) above 48 units per/liter, thrombocytopenia below 150,000 counts per microliter, hypercholesterolemia above 200 mg/dl and hyper triglycerides above 1.7 millimoles per liter.

### Data source/data collection

At each data collection period, we measured participants’ BMIs using clinic-based weighing scales and Stadiometers, blood pressure (by auscultation technique using mercury sphygmomanometer, first and fourth Korortkoff sounds were taken as systolic and diastolic values, respectively – women rested for, at least, five minutes, seated comfortably in upright position in quiet rooms, not talking, eating or watching television with feet resting flat on floor during the procedure. Urine protein was measured (using meditest combi 10 dipsticks) and a general physical examination for symptoms and signs conducted. In addition, blood samples were collected for laboratory tests which included blood platelet estimation, liver function tests (AST, ALT), renal function assessment (serum urea, creatinine and uric acids), glucose tolerance test (random and fasting blood sugar) and serum lipids (cholesterols and triglycerides). Laboratory investigations were performed by laboratory analists based on universal laid down protocol and standard practices used in all public tertiary hospitals in Nigeria (Supplement Table 1).

### Sample size

Based on the existing knowledge that 10% of pregnant women develop HDPs, we estimated that 153 women were required to participate in the study, including a 10% potential non-response (at 5% alpha level and power of 80%). The following formular was used: n = (z^2^) p(1–p)/d2, where n = required sample size, z = z statistic for the level of confidence (1.96), p = expected prevalence (10%) and d is the allowable error (0.0025). Because this study carries minimal risk to the participants, we subsequently enrolled 410 with HDPs.

### Risk of bias

In order to reduce biases, participants were recruited from a similar population of women giving birth in these facilities. Since in Nigeria women go to tertiary hospitals for antenatal and delivery services based on their places of residence and socio-economic status (not based on referrals), a possible selection bias in the direction of high risk confers by socio-economic status cannot be rule-out. Recruitment proceeded independently and concurrently in all facilities until the desired sample size was reached. Case identification was done by specially trained and experienced midwives using standard diagnostic criteria. Outcome assessors were not aware of clients’ categorization into various HDP categories. The results of medical and laboratory investigations were entered in the electronic data capturing platform by trained research assistants as soon as the data were available to reduce the incidence of missing data. The hard copies of all medical and laboratory results were retained as source documents at the facilities for future reference when necessary.

### Data analysis plan/statistical methods

Frequencies, percentages and simple proportion (mean and standard deviations) were used to describe participants’ obstetrics, demographics, and other variables by HDP group. Women with eclampsia were included in the pre-eclampsia sub-classification, and women with pre-existing hypertension (chronic hypertension) were not analyzed beyond their proportion in the study population. We used bar charts to illustrate trends in magnitudes of blood pressure response since delivery. Trends to blood pressure normalization since delivery were analyzed, using proportions and percentages, separately for chronic hypertension, gestational hypertension and pre-eclampsia. Univariable and multivariable logistic regression analysis was used to determine factors that predict the presence or absence of hypertension at one year after delivery. Possible predictor variables were hypothesis-driven selected based on previous literature and clinical knowledge and included age, BMI at baseline, random blood glucose at baseline, gestational age at onset of HDP and at delivery, systolic and diastolic blood pressure at baseline. The data were analyzed using SPSS IBM version 25.0.

### Missing data

During every follow-up period, clinical and laboratory information were obtained and entered immediately into the electronic data capturing platform to minimize instances of missing data. Cases of missing data occurred when subjects failed to report for data collection periods. We assume that data were missing completely at random. Therefore, complete case analysis was performed, such that for any data collection period, only clients that have reported and provided complete information were analyzed.

## Ethical approval

The study was approved by the Population Council’s institutional review board in New York (protocol no. 810), National Health Research Ethics Committee (NHREC) at the Federal Ministry of Health and by the institutional review boards at all the participating hospitals.

## Results

**Figure 2:**
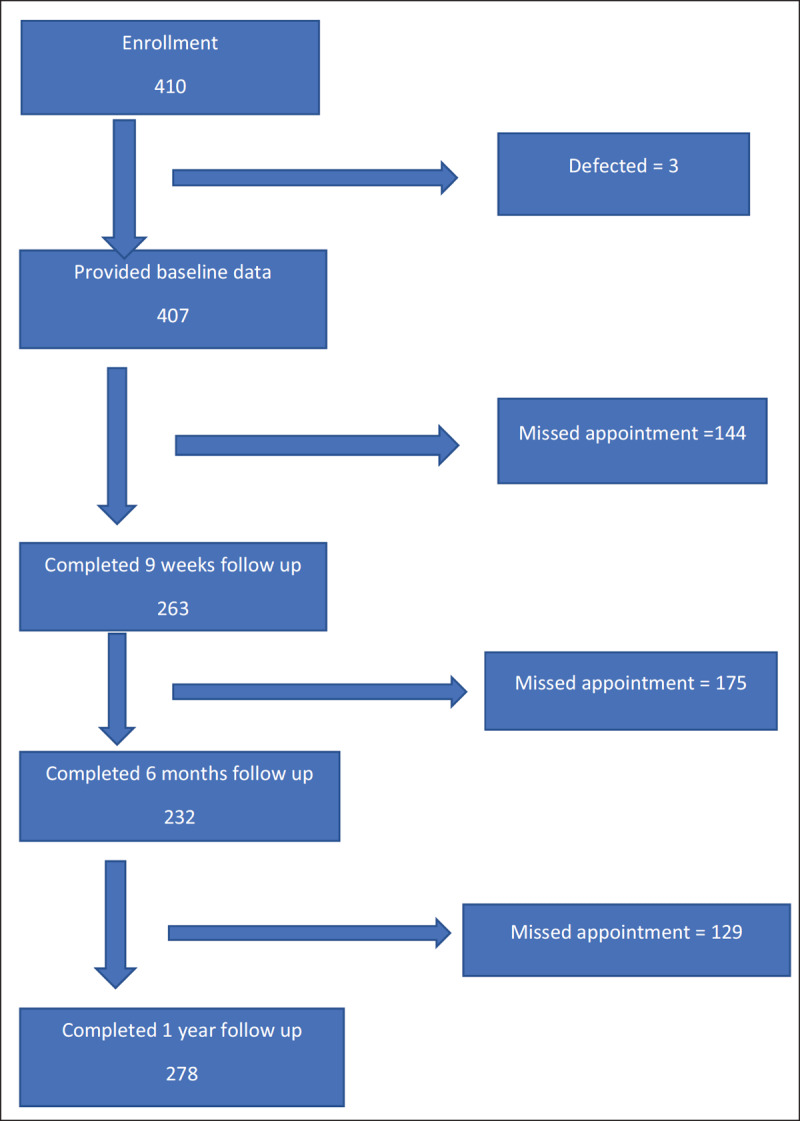
Flowchart of Participants enrollment and follow up process.* * The loss to follow up were actually cases of missed appointment as specific time period. Also, some of the missed appointees at 9 weeks reported at 6 months and or at 1 year follow up.

The recruitment and follow up of study participants are depicted in Figure 2. Of the 410 women with HDPs who were enrolled, 407 provided baseline data and 263, 232 and 278 women followed up at nine weeks, six months and one year, respectively (a 68% follow-up rate at one year). Table 1 presents the baseline characteristics of the participants. The majority of women (60%, n = 247) had antenatal care, 3% of whom registered in the first trimester. The mean gestational ages at the onset of HDP and delivery were 33 weeks (standard deviation [sd] 8.8) and 36 (sd 4.1) weeks, respectively. The mean BMI at booking among those who received antenatal care was 28 kg/m^2^ (sd 7.7). The perinatal death rate was 9% (35): 7% (29) and 2% (6) for stillbirths and early neonatal deaths respectively.

**Table 1:** Socio-demographic and obstetric characteristics of the of 410 women with HDPs enrolled in the study.

Variables	Number (%)	Mean (SD)	GHT	CHT	PE	EC

Mean age (SD)	–	28.9 (6.4)	33.3 (28.9)	35.2 (6.8)	28.4 (5.7)	24.6 (6.3)
Mean BMI at booking (SD)	–	28.8 (7.7)	31.6 (8.5)	31.7 (11.7)	27.7 (6.2)	24.9 (4.2)
*Parity (N = 366)*						
Para 0 n(%)	78 (21.4)	–	9 (12.0)	1 (3.0)	45 (22.5)	23 (40.3)
Para 1 – 3 n(%)	200 (54.8)	–	51 (68.0)	14 (42.4)	108 (54)	27 (47.4)
≥para 4 n(%)	87 (23.8)	–	15 (20.0)	18 (54.6)	47 (23.5)	7 (12.3)
*Booking status n(%)*						
Booked	247 (60.2)		60 (80.0)	23 (70.0)	117 (58.5)	21 (36.8)
Unbooked	163 (39.8)		15 (20.0)	10 (30.0)	83 (41.5)	36 (63.2)
Number enrolled per HDP types	–	–	76 (18.5)	47 (11.5)	205 (50.0)	82 (20.0)
Number reviewed at 9 weeks per HDP types	–	–	54 (20.5)	24 (9.0)	143 (54.5)	42 (16.0)
Number reviewed at 6 months per HDP types	–	–	44 (19.0)	24 (10.5)	122 (52.5)	42 (18.0)
Number reviewed at 1 year per HDP types	–	–	69 (25)	42 (15)	136 (49)	31 (11)
Mean Gestational age at booking	–	–	24.9 (6.7)	23.8 (4.6)	23.1 (6.3)	23 (6.7)
*Distribution of GA at booking n(%)*						
≤12 weeks	14 (3.4)	–	3 (4.0)	0 (0.0)	7 (3.5)	2 (3.5)
13 – 20 weeks	73 (17.8)	–	14 (18.7)	6 (18.2)	43 (21.5)	6 (10.5)
>20 weeks	323 (78.8)	–	58 (77.3)	27 (81.8)	150 (75)	49 (86)
Mean Gestational age at onset of HDP	–	33.2 (8.8)	36.9 (4.1)	–	33 (8.5)	34.5 (6.5)
*GA distribution of onset of HDP*						
≥ 34 weeks	239 (65.5)	–	61 (81.3)	–	122 (61)	42 (73.7)
<34 weeks	126 (34.5)	–	11 (14.7)	–	78 (39)	15 (26.3)
Mean Gestational age at delivery (SD)	–	36.5 (4.1)	38.7 (1.9)	36.1 (3.9)	36 (4.3)	35.5 (4.3)
*Distribution of GA at delivery n(%)*						
>=37 weeks	265 (64.6)	–	67 (89.3)	18 (54.5)	103 (51.5)	31 (54.4)
<37 weeks	145 (35.4)	–	8 (10.7)	15 (45.5)	97 (48.5)	26 (45.6)
*Mode of Delivery n(%)*						
Spontaneous Vaginal Delivery	165 (45.1)	–	42 (56)	18 (54.5)	82 (41)	23 (40.4)
Assisted Vaginal Delivery	6 (1.6)	–	2 (2.7)	0 (0.0)	3 (1.5)	1 (1.8)
Elective Caesarean section	19 (5.2)	–	6 (8)	2 (6.1)	11 (5.5)	0 (0.0)
Emergency Caesarean section	176 (48.1)	–	25 (33.3)	13 (39.4)	104 (52)	33 (57.8)
*Apgar score at 1 min n(%)*						
Apgar >= 7	224 (61.4)	–	58 (77.3)	20 (60.6)	117 (58.5)	29 (50.9)
Apgar < 7	141 (38.6)	–	17 (22.7)	13 (39.4)	83 (41.5)	28 (49.1)
*Apgar score at 5 min n(%)*						
Apgar >= 7	308 (84.4)	–	72 (96.0)	29 (87.9)	163 (81.5)	44 (77.2)
Apgar < 7	57 (16.6)	–	3 (4.0)	4 (12.1)	37 (18.5)	13 (22.8)
*Perinatal deaths n(%)*						
Stillbirths	29 (70.7)	–	2 (66.7)	1 (33.3)	20 (69)	6 (100.0)
Early neonatal deaths	12 (29.3)	–	1 (33.3)	2 (66.7)	9 (31)	0 (0.0)
Proportion of HDPs with BP ≥150/100 mmHg within five days of delivery on anti-hypertensive medications	33%	–				

HDP = Hypertensive disorders in pregnancy, SD = standard deviation, GHT = Gestational hypertension, CHT = Chronic hypertension, PE = Pre-eclampsia, EC = Eclampsia, GA = gestational age, BP = Blood pressure.

**Figure 3:**
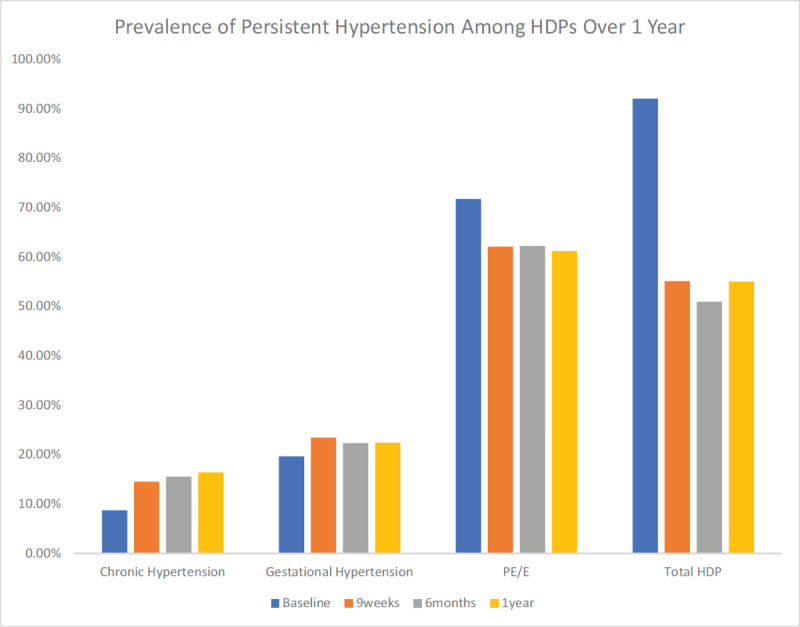
Prevalence of persistent hypertension (BP >140/90 mmHg) based on HDP sub-types and total over one year of follow up. HDPs = Hypertensive disorders in pregnancy; PE/E = Pre-eclampsia/eclampsia.

**Figure 4:**
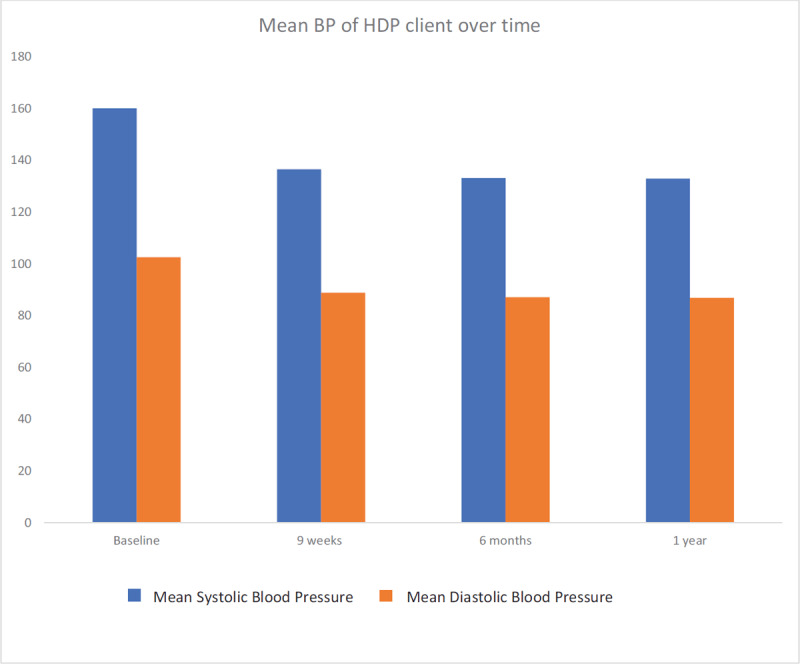
Trend in mean systolic and diastolic blood pressure over one year of follow up. BP = Blood pressure, HDP = Hypertensive disorders in pregnancy.

The proportion of women with persistent hypertension (systolic of ≥140 mmHg and or diastolic of ≥90 mmHg) at nine weeks, six months and one year after delivery is shown in Figure 3. For women with gestational hypertension, persistent hypertension at nine weeks, six months and one year occurred in 23% (95% CI; 6.6–52.6), 22% (95% CI; 8.3–36.3) and 22%, (95% CI; 5.6–54.4) respectively. Women with pre-eclampsia/eclampsia had a much higher prevalence: 62% (95% CI; 47.9–76), 62% (95% CI; 52.5–71.9) and 61% (95% CI; 40.6–77.8), respectively. The mean systolic and diastolic blood pressure (Figure 4) declined progressively from delivery baseline values of 160 mmHg and 102.5 mmHg respectively to a one-year mean values of 132.9 mmHg and 86.9 mmHg, respectively. Within the first five postpartum days, only 33% of those with high blood pressure were on anti-hypertensive therapy.

Table 2 shows the results of univariable and multivariable logistic regression to assess the association between 13 possible risk factors for the presence of persistent hypertension one year after delivery among women with gestational hypertension and pre-eclampsia/eclampsia (chronic hypertension in pregnancy excluded in the determinants). Of these predictors, maternal age, BMI, systolic and diastolic blood pressure at delivery significantly predicted the presence of persistent hypertension at one year in univariate analysis [ORs =1.11/year (95% CI; 1.06–1.15), 1.03/kg/m^2^ (95% CI; 1.00–1.07), 1.01/mmHg (95% CI; 1.00–1.02) and 1.02/mmHg (95% CI; 1.00–1.03) respectively]. In the multivariable analysis, only maternal age and BMI at delivery were independent predictors and increased the risk of persistent hypertension at one year after delivery following HDPs [adjusted OR 1.07/year (95% CI; 1.02–1.13) and 1.06/kg/m^2^ (95% CI; 1.01–1.1)] respectively.

**Table 2:** Univariable and multivariable logistic regression analysis of factors associated with persistent hypertension among 254 women with HDPs (excluding chronic hypertension) at one year after delivery.

Variable	Univariate		Multivariate	

	OR (95% CI)	P-value	OR (95%CI)	P-value

Age	1.11 (1.06–1.15)	0.000	1.07 (1.02–1.13)	0.011
BMI at baseline	1.03 (1.00–1.07)	0.147	1.06 (1.01–1.10)	0.010
Random blood glucose at baseline	0.99 (0.90–1.09)	0.869	0.92 (0.80–1.06)	0.258
Gestational age at onset of HDP	0.97 (0.94–1.00)	0.055	0.99 (0.96–1.05)	0.948
Gestational age at delivery	0.97 (0.92–1.04)	0.513	0.98 (0.91 –1.07)	0.729
Systolic BP at baseline	1.01 (1.00–1.02)	0.030	1.01 (0.99–1.03)	0.633
Diastolic BP at baseline	1.02 (1.00–1.03)	0.067	1.01 (0.98–1.04)	0.421
Diagnosis of gestational hypertension	0.25 (0.09–0.71)	0.009	0.71 (0.44–1.15)	0.164
Diagnosis of pre-eclampsia	0.20 (0.08–0.52)	0.001		
Raised creatinine >90 mmol/L	1.04 (0.90–1.20)	0.061	1.06 (0.90–1.25)	0.477
Raised uric Acid >446 mmol/L	1.01 (0.95–1.06)	0.797	1.03 (0.96–1.10)	0.471
Raised Triglycerides>1.7 mmol/l	0.99 (0.88–1.11)	0.871	0.95 (0.80–1.12)	0.520
Raised cholesterol>200 mg/dL	0.99 (0.99–1.00)	0.634	0.99 (0.99–1.00)	0.594

* p < 0.05, OR = Odd ratios, BP = Blood pressure, HDP = Hypertensive disorders in pregnancy.

## Discussion

To date this cohort study of women with HDP recruited and followed up from delivery until one year afterward is the largest and longest conducted in a sub-Saharan African setting. This study observed high rates of persistent hypertension regardless of HDP subtype (21% and 61% of women with gestational hypertension and pre-eclampsia, respectively). This is in line with findings from other studies within sub-Sahara Africa in Nigeria, Cameroon, Uganda and Sudan [10,11,12,13,14], which reported rates between 25.5% and 35.6% six weeks to six months after delivery of HDPs-complicated pregnancies.

Rates of persistent hypertension in pre-eclampsia of 39% and 18% at three months and two years postpartum, respectively, and higher mean blood pressure among pre-eclamptic cohort six months after delivery have also been reported in the Netherlands [5,19]. Switzerland reported 57.4% six to twelve weeks after delivery [6], while United States reported 21% at six weeks postpartum [20]. Japanese women required more than two months after delivery for normalization of blood pressure following gestational hypertension and pre-eclampsia [21]. While these studies looked at either pre-eclampsia or gestational hypertension, the patterns and magnitude of prevalence of persistent hypertension across HDP sub-types and time from delivery appear common in sub-Saharan African women and others.

Although hypertension persists up to one year in many women in our study, the proportion and severity of hypertension progressively declined after delivery. The proportion of persistent hypertension, mean systolic and diastolic blood pressure progressively declined after delivery up to one year. This trend was also reported in a Cameroonian study with hypertension prevalence declining from 42.8%, 27.8% and 14.8% at six weeks, three months and six months postpartum, respectively [11]. As previously noted elsewhere [5], our study showed a significant proportion of women with persistent hypertension following HDPs will not resolve after a year postpartum. In addition to a progression to develop chronic hypertension for previously normotensive women [7,8,31], this could also be explained by an underdiagnosis of pre-pregnancy chronic hypertension due to a lack of blood pressure measurements prior to pregnancy or a masking because of the first and second trimester psychiologic blood pressure reduction [7].

If women with HDPs are not monitored long enough until blood pressure normalized or referred to continued medical practitioners’ care, hypertension can persist unnoticed and increase the risk of adverse cardiovascular and renal complications (22% prevalence of chronic kidney diseases has been reported among people with undiagnosed hypertension) [22,23,24,26]. Notably, we observed from unpublished data on adherence to quality of care for women with HDPs that only one-thirds of women with hypertension in the first five days of delivery were on anti-hypertensive treatment. Although we did not assess this measure of compliance to treatment beyond this period, given previous evidence and the health care context [27,28], it is unlikely to expect compliance would have improved over time.

Maternal age and BMI were predictors of persistent hypertension, as were observed in prior studies [11,18,19], and were expected based on the known associations between BMI, age and chronic hypertension. These have important clinical and public health implication given the mean age (28.9 years) and BMI (28.8 kg/m^2^) among our cohort at ANC booking. While acknowledging that only 3% of our cohort registered for ANC within the first trimester when BMI measurement could have been more reliable, it is still concerning given the overweight and obesity prevalence among non-pregnant Nigerian women of 16.2% and 6.6%, respectively [29]. The relationships between abnormal pre-pregnancy BMI and adverse feto-maternal outcomes have long been established and this finding strengthens a call for routine pre-conception care services [30], risk factors-based focused ANC and lifestyle modification for women in sub-Saharan Africa. BMI is a modifiable risk factor and women can be counselled to maintain healthy BMIs after delivery, and before future conceptions to reduce the risk of complications [32], including subsequent persistent hypertension as shown in this study.

### Study strengths

Our study was able to follow up nearly 70% of participants at one year postpartum; a reflection of extensive investments to retain women in the study within this context. This retention rate was also commendable based on recommended follow up rates between 50 – 80% for cohort studies [15,16,17]. To our knowledge, this is the largest cohort of women with HDPs followed up the longest in African settings (previous single-center studies in Cameroon, Nigeria, Sudan and Ugandan were for between six weeks to six months) [10,11,12,13,14]. This study enrolled women from eight tertiary health facilities spread across Nigeria ensuring diversity of patients and settings.

### Limitations

Despite our reasonable follow-up rate, the missing 32% of the women may have introduced limitations to the generalizability of the findings. However, analysis of sociodemographic and obstetrics characteristics between women who completed one year follow up and those lost (missed appointments) did not show difference between the two groups (Supplement Table II). The lack of information of BP prior to pregnancy almost certainly lead to underestimation of chronic hypertension present before pregnancy. We used 4^th^ Korotkoff as diastolic BP which shows poor reproducibility in studies [36]. Also, as women of higher socio-economic status (often of higher maternal age and BMIs compared to general maternal age) self-select themselves for routine ANC and delivery care at tertiary hospitals in Nigeria, this might may have caused overestimation of both determinants and outcomes.

### Clinical and research implications

All women with HDPs, irrespective of the sub-type, should be managed and followed up until hypertension is normalized either physiologically or supported by appropriate anti-hypertensive treatment [1,2]. In this study, over 60% of women with continuously elevated high blood pressure within the first week after delivery discontinued anti-hypertensive treatment. If this pattern is not reversed, the long-term cardiovascular and other poor health outcomes could be enormous. A large systematic review and meta-analysis has reported after pre-eclampsia complicated pregnancies a threefold higher risk for chronic hypertension (RR 3.70, 95% CI 2.70–5.05) after 14.1 years weighted mean follow-up, a two-fold higher risk for ischemic heart diseases after 11.7 years (RR 2.16, 95% CI 1.86–2.52) nearly twofold higher risk of stroke (RR 1.81, 95% CI 1.45–2.27) after 10.4 years [33].

The rising burden of cardiovascular diseases such as ischemic heart disease and stroke in sub-Saharan Africa underscores the importance of early recognition of women at risk [23,25]. As both hypertension and pre-eclampsia are independent risk factors for cardiovascular diseases [24,28], adequate management of these risk factors can improve the future health of these women and reduce the burden to the health system. As such, HDP need to be included in guidelines for public health and individual health cardiovascular risk management (CVRM) strategies and risk models for women future cardiovascular health [24]. Importantly, beyond inclusion of postpartum management for women with HDPs into existing guideline, effective implementation will be an essential challenge to overcome, especially in sub-Saharan African setting. Therefore, ongoing monitoring and evaluation would be necessary.

## Conclusion

A substantial percentage of women who have had pregnancies complicated by hypertensive disorders have persistent hypertension beyond puerperium, and up to one year postpartum. Therefore, women with HDP-complicated pregnancies need regular postpartum evaluation until blood pressure is normalized or referral for further medical or primary care physician follow up including lifestyle modification, to reduce persistent hypertension and subsequent CVDs risk after delivery. These are strategies that may be effective, but still need to be evaluated for effectiveness after implementation on the reduction of HPD in a next pregnancy and long-term hypertension and CVD. Given the persistent burden of cardiovascular disease in pregnancy and pre-mature deaths of women globally, this will support the attainment of the Sustainable Development Goals.

## Data Accessibility Statements

All data are available on request to the first author.

## Additional File

The additional file for this article can be found as follows:

10.5334/gh.854.s1 Supplement. Tables I and II.

## References

[1] Magee LA, Dadelszen PV, Stones W, Mathai M. An Evidence-based guide to monitoring, prevention and management. The FIGO textbook of pregnancy hypertension. 2016; 5–6

[2] Brown MA, Magee LA, Kenny LC, et al. Hypertensive disorders of pregnancy ISSHP Classification, Diagnosis, and Management Recommendations for International Practice. Hypertension. 2018; 72: 24–43. DOI: 10.1161/HYPERTENSIONAHA.117.10803 29899139

[3] Oladapo OT, Adetoro OO, Ekele BA, et al. Nigeria Near-miss and Maternal Death Surveillance Network. When getting there is not enough: A nationwide cross-sectional study of 998 maternal deaths and 1451 near-misses in public tertiary hospitals in a low-income country. Br J Obstet Gynaecol. 2015. DOI: 10.1111/1471-0528.13450 PMC5016783 25974281

[4] Rosemary T, Patrick O’, Asma K. Current best practices in the management of hypertensive disorders in pregnancy. Integrated Blood Pressure Control. 2016; 9: 79–94. DOI: 10.2147/IBPC.S77344 27555797 PMC4968992

[5] Berks D, Eric APS, Marek M, Willy V. Resolution of hypertension and proteinuria after pre-eclampsia. Obstet and Gynecol. 2009; 114(6): 1307–1314. DOI: 10.1097/AOG.0b013e3181c14e3e 19935034

[6] Agnes D, Wuerzner G, Ponte B, et al. Prevalence of hypertensive phenotypes after preeclampsia: Prospective cohort study. Hypertension. 2018; 71: 103–109. DOI: 10.1161/HYPERTENSIONAHA.117.09799 29133363

[7] Schwartz CL, McManus RJ. What is the evidence base for diagnosing hypertension and for subsequent blood pressure treatment targets in the prevention of cardiovascular disease? BMC Medicine. 2015; 13: 256. DOI: 10.1186/s12916-015-0502-5 26456709 PMC4601133

[8] Leening MJG, Ikram MA. Primary prevention of cardiovascular disease: The past, present, and future of blood pressure- and cholesterol-lowering treatments. PLoS Med. 2018; 15(3): e1002539. DOI: 10.1371/journal.pmed.1002539 29558473 PMC5860691

[9] Gregory AR, Mark DH, Andrew EM, et al. Global and Regional Patterns in Cardiovascular Mortality from 1990 to 2013. Circulation. 2015; 132: 1667–1678. DOI: 10.1161/CIRCULATIONAHA.114.008720 26503749

[10] Fadalallah ZM, Elhassan EM, Rayis DA, Abdullahi H, Adam I. Prospective cohort study of persistent hypertension following pre-eclampsia at Medani Hospital, Sudan. Intl J Gynecol Obstet. 2016; 134: 66–68. DOI: 10.1016/j.ijgo.2015.11.014 26975905

[11] Kaze FF, Njukeng FA, Andre-Pascal K, et al. Post-partum trend in blood pressure levels, renal function and proteinuria in women with severe preeclampsia and eclampsia in Sub-Saharan Africa: A 6-months cohort study. BMC Pregnancy and Childbirth. 2014; 14: 134. DOI: 10.1186/1471-2393-14-134 24712704 PMC4004513

[12] Nakimuli A, Elliott AM, Kaleebu P, Moffett A, Mirembe F. Hypertension Persisting after Pre-Eclampsia: A Prospective Cohort Study at Mulago Hospital, Uganda. PLoS ONE. 2013; 8(12): e85273. DOI: 10.1371/journal.pone.0085273 24392003 PMC3877387

[13] Ndayambagye EB, Nakalembe M, Kaye DK. Factors associated with persistent hypertension after puerperium among women with preeclampsia/eclampsia in Mulago hospital, Uganda. BMC Pregnancy and Childbirth. 2010; 1471–2393/10/12. DOI: 10.1186/1471-2393-10-12 PMC2848130 20222993

[14] Olagbuji B, Ezeanochie M, Ande A, Okonkwo C. Prevalence and risk factors for persistent hypertension after the puerperium in pregnancies complicated with hypertensive disorders. J Obstet Gynaecol. 2012 8; 32(6): 529–32. DOI: 10.3109/01443615.2012.689891 22779954

[15] Kristman V, Manno M, Côté P. Loss to Follow-Up in Cohort Studies: How Much is Too Much? European Journal of Epidemiology. 2004; 19(8): 751–60. DOI: 10.1023/B:EJEP.0000036568.02655.f8 15469032

[16] Lohr SL. Nonresponse. In: Lohr SL (ed.), Sampling: design and analysis. Pacific Grove: Duxbury Press, 1999. 255–287.

[17] Altman DG. Statistics in medical journals: Some recent trends. Stat Med. 2000; 19: 3275–3289. DOI: 10.1002/1097-0258(20001215)19:23<3275::AID-SIM626>3.0.CO;2-M 11113959

[18] Hwang J, Park S, Oh S, et al. The risk factors that predict chronic hypertension after delivery in women with a history of hypertensive disorders of pregnancy. Medicine. 2015; 94(42): e1747. DOI: 10.1097/MD.0000000000001747 26496291 PMC4620832

[19] van Rijn BB, Nijdam ME, Bruinse HW, et al. Cardiovascular disease risk factors in women with a history of early-onset preeclampsia. Obstet Gynecol. 2013; 121(5): 1040–8. DOI: 10.1097/AOG.0b013e31828ea3b5 23635741

[20] Levine LD, Nkonde-Price C, Limaye M, Srinivas SK. Factors associated with postpartum follow-up and persistent hypertension among women with severe preeclampsia. J Perinatol. 2016; 36(12): 1079–1082. DOI: 10.1038/jp.2016.137 27583396 PMC5480377

[21] Mikami Y, Takagi K, Itaya Y, et al. Post-partum recovery course in patients with gestational hypertension. J Obstet Gynecol Res. 2014; 40(4): 919–25. DOI: 10.1111/jog.12280 24428339

[22] World Health Organization. Cardiovascular diseases. Geneva, Switzerland: World Health Organization. http://www.who.int/mediacentre/factsheets/fs317/en/.

[23] Suzan H, Wim G, Milena P. Trends in cardiovascular diseases and associated risks in sub-Saharan Africa: A review of the evidence for Ghana, Nigeria, South Africa, Sudan and Tanzania, The Aging Male. 2019; 22(3): 169–176, DOI: 10.1080/13685538.2019.1582621 30879380

[24] American Heart Association, American Stroke Association. Guidelines for the Prevention of Stroke in Women: A Statement for Healthcare Professionals from the American Heart Association/American Stroke Association. Stroke. 2014; 45: 1545–1588. DOI: 10.1161/01.str.0000442009.06663.48 24503673 PMC10152977

[25] Jean JN, Jean JB, Ulrich FN, et al. The burden of hypertensive disorders in Africa: A systematic review and meta-analysis. J Clin Hypertens. 2019; 21: 479–488. DOI: 10.1111/jch.13514 PMC8030504 30848083

[26] Deidra CC, Laura CP, Edgar RM, et al. Prevalence of Chronic Kidney Disease in Persons with undiagnosed or prehypertension in the United States. Hypertension. 2010; 55(5): 1102–1109. DOI: 10.1161/HYPERTENSIONAHA.110.150722 20308607 PMC2880533

[27] Elysia La, Miriam R, Godfrey MM, Redempta M, Margaret EK. Missed opportunities to improve the health of postpartum women: High rates of untreated hypertension in rural Tanzania. Matern Child Health J. 2017; 21(3): 407–413. DOI: 10.1007/s10995-016-2229-0 28120288 PMC5357453

[28] Boateng D, Wekesah F, Browne JL, et al. Knowledge and awareness of and perception towards cardiovascular disease risk in sub-Saharan Africa: A systematic review. PLoS ONE. 2017; 12(12): e0189264. DOI: 10.1371/journal.pone.0189264 29232703 PMC5726714

[29] Ghose B, Yaya S. Media use and excess body weight among women in Nigeria: A cross-sectional study. BMJ Open. 2018; 8: e020802. DOI: 10.1136/bmjopen-2017-020802 PMC6045715 29982206

[30] Joice MV, Jusciele BM, Claudia GM, et al. Maternal and fetal outcomes in pregnancies complicated by overweight and obesity. Reproductive Health. 2016; 13: 100. DOI: 10.1186/s12978-016-0206-0 27567898 PMC5002321

[31] Reem M, Sana A, Anu G, Rocco CV. A Comprehensive Review of Hypertension in Pregnancy. Journal of Pregnancy. Volume 2012. DOI: 10.1155/2012/105918 PMC3366228 22685661

[32] Adane AA, Mishra GD, Tooth LR. Adult Pre-pregnancy Weight Change and Risk of Developing Hypertensive Disorders in Pregnancy. Paediatr Perinat Epidemiol. 2017; 31(3): 167–175. DOI: 10.1111/ppe.12353 28386955

[33] Bellamy L, Casas JP, Hingorani AD, Williams DJ. Pre-eclampsia and risk of cardiovascular disease and cancer in later life: Systematic review and meta-analysis. BMJ. 2007; 335(7627): 974. DOI: 10.1136/bmj.39335.385301.BE 17975258 PMC2072042

[34] Angela T, Cristiano F. Diagnosis of hypertensive disorders in pregnancy: An update. Journal of Laboratory and Precision Medicine. DOI: 10.21037/2019.11.04

[35] Magee LA, Sharma S, Nathan HL, et al. The incidence of pregnancy hypertension in India, Pakistan, Mozambique, and Nigeria: A prospective population-level analysis. PLoSMed. 16(4) e1002783. DOI: 10.1371/journal.pmed.1002783 PMC6461222 30978179

[36] Arie F, Joris AMV, Gert AVM, Hein WB, Gerard HAV. The fourth sound of Korotkoff in pregnancy: A myth? The Lancet. 1996; 347: 841. DOI: 10.1016/S0140-6736(96)90926-5 8622381

