# Supplementary material for: Persistent Hypertension Up to One Year Postpartum among Women with Hypertensive Disorders in Pregnancy in a Low-Resource Setting: A Prospective Cohort Study
Source: Glob Heart. 2021 Sep 9;16(1):62. doi: 10.5334/gh.854 (PMC8428291; doi:10.5334/gh.854)
Supplement: Supplement. — Tables I and II. [file gh-16-1-854-s1.pdf]

SUPPLEMENT TABLE I: Laboratory protocol and standard for public tertiary hospitals in Nigeria

S/N	ANALYTE/PARAMETER	ANALYTICAL PRINCIPLE	ANALYSER/EQUIPMENT/PLATFORM
1	Glucose	Trinder's Method: Glucose Oxidase	Roche C311 and Abbott C4000
2	Sodium	Ion Selective Electrode(**ISE**)	Notek EL 3000
3	Potassium	ISE	Notek EL 3000
4	Chloride	ISE	Notek EL 3000
5	Bicarbonate	ISE	Notek EL 3000
6	Urea	Berthelot's Method: Urease Method	Roche C311 and Abbott C4000
7	Creatinine	Modified Jaffe Kinetic Method	Roche C311 and Abbott C4000
8	Uric acid	Uricase method	Roche C311 and Abbott C4000
9	Serum Total Calcium	Cresophthalein Complexone	Roche C311 and Abbott C4000
10	Inorganic Phosphate	Ammonium Phosphomolybdate method	Roche C311 and Abbott C4000
11	Albumin	Bromocresol Green (BCG)	Roche C311 and Abbott C4000
12	AST	Spectrophotometric measurement of NADH coupled Oxoacid formation	Roche C311 and Abbott C4000
13	ALT	Spectrophotometric measurement of NADH coupled Oxoacid formation	Roche C311 and Abbott C4000
14	ALP	Spectrophotometric Using NPP as substrate	Roche C311 and Abbott C4000
15	Total Bilirubin	Modified Diazo(Malloy Evelyn) Method	Roche C311 and Abbott C4000
16	Direct Bilirubin	Modified Diazo(Malloy Evelyn/Jendrassic Groff) Method	Roche C311 and Abbott C4000

SUPPLEMENT TABLE II): Distribution of Sociademographic and Obstetric Characteristic of HPD Clients that Were Followed up versus Loss to Follow up at one Year

**Variables**	**Followed up to 1 year**	**Loss to followup at one year**
Mean age (SD)	29.8(6.2)	29.1(19.9)
Mean Gestational age at onset of HDP (SD)	33(9.9)	33.5(7.3)
Mean Gestational age at booking (SD)	23.4(6.8)	24.3(5.6)
Category of HDP		
Chronic Hypertension n(%)	15(7.54)	18(11.1)
Gestational hypertension n(%)	52(26.1)	20(12.4)
Pre-eclampsia n(%)	107(53.8)	92(56.8)
Eclampsia n(%)	25(12.6)	32(19.8)
Mean BMI at baseline (SD)	29.2(7.9)	28.2(7.5)
Mean BMI at 1 year (SD)	28.7(7.2)	___
Mean systolic BP at baseline (SD)	158.8(20.8)	161.7(23.3)
Mean systolic BP at 1 year (SD)	133.3(26.6)	___
Mean diastolic at baseline (SD)	137.1(23.4)	135(29.3)
Mean distolic at 1 year (SD)	86.1(18.3)	___
